# The shaping of pharmaceutical governance: the Israeli case

**DOI:** 10.1186/2045-4015-3-16

**Published:** 2014-05-27

**Authors:** Philip Sax

**Affiliations:** 1 PHARMA Drug Bulletin, Centre for Drug Studies, Jerusalem 9262807, Israel

**Keywords:** Health insurance, Pharmaceutical governance, Pharmaceutical policy, Reimbursement, Pricing, Israel

## Abstract

This article focuses on governance of the pharmaceutical sector in Israel. It traces the relationships between the state, industry, and sick funds from before the establishment of National Health Insurance (NHI) in 1995 to the beginning of this decade, in particular as they have grappled with the challenge of making national formulary decisions in a rational manner. Subsequent to the introduction of NHI there have been shifts in the modes and mix of governance. This research shows empirically that a relatively complex mix of hierarchical and network modes of governance can be successfully established over an extended period of time when flexibility is maintained through the implementation process. The system for defining and updating a standard basket of health services has coped well with the challenge of managing a range of difficult and potentially volatile stakeholder relationships in the pharmaceutical sector and of distancing ministers from controversies of funding and listing decisions. Government has succeeded in containing drug costs whilst still maintaining a basket of reimbursable drugs that, from an international perspective, is comprehensive and technologically advanced.

On the other hand, network arrangements appear to have delayed the introduction of suitable accountability relationships and hindered their development. The state has traditionally played an intermediary role between unavoidable corporate interests of industry and sick funds, with little transparency and to the detriment of more pluralistic access to decision making. Governance arrangements in Israel appear to limit the potential and incentive of the state and the sick funds to realize their potential countervailing powers in subsidy and pricing decisions.

## 

This article focuses on governance of the pharmaceutical sector in Israel. It traces the relationships between the state, industry, and sick funds from before the establishment of National Health Insurance (NHI) in 1995 to the beginning of this decade, in particular as they have grappled with the challenge of rationalizing pharmaceutical provision. Mainly as a result of the introduction of NHI there have been shifts in the modes and mix of governance over an extended time period.

The literature on pharmaceutical governance has focused on State actors such as single-payer national health systems and in the main on the regulatory tensions with the interests of the research-based multinational pharmaceutical industry [1-3]. In the case of Israel, the role of non-State actors, that is of publicly-funded private (not-for-profit) sick funds - as well as a pharmaceutical sector dominated by generic industry interests - are special features that could add institutional diversity to the international literature on governance regimes in the health and pharmaceutical sectors. As such, the paper aims to throw light on the multi-levelness of policy-making and governance.

Israel’s health care system represents an interesting laboratory for policy experiments, as it shares certain key characteristics with many European countries, such as competing sick funds and a commitment to principles of equity and solidarity, though it is not encumbered by supranational regulatory requirements common to the EU [4]. Since the enactment of NHI, Israel has had almost 20 years of consistent and cumulative experience with the active management of an explicitly defined drug benefits scheme and has established a distinct culture and modus operandi that are worthy of study.

Within the context of the governance of the pharmaceutical sector I have chosen to focus on the making of national formulary decisions. A core government policy objective is cost containment, an issue in which the interests of government, sick funds and a generic-based industry coincide, at least to some extent. In the NHI era Israel has achieved long-term success in cost-containment [5], with the key factors including: a tight health budgetary framework within NHI; a small number of dominant sick funds; and comprehensive policies for generic medicines [6,7]. Israel has had success in managing pharmaceutical expenditures while maintaining universal access to medically necessary pharmaceuticals since the introduction of NHI.

On the other hand, the issue of equity has received belated attention [6]. Only about 60% of pharmaceutical expenditure in Israel is funded via NHI, with the balance consisting of patient co-payments as well as purchases of drugs (or their specific indications) not covered by the NHI including OTC medicines.

The first section Concepts and theoretical issues of the paper introduces concepts related to the shift in the governance of the pharmaceutical sector, including the theory of countervailing forces. The second section The Israel case presents the Israeli case. The third section Discussion discusses the successes and failures of the Israeli approach to date and suggests ways forward. An Appendix presents a descriptive analysis of the Israeli pharmaceutical industry context, focusing on the local generic-based industry and its role in the policy network.

## Concepts and theoretical issues

The literature on trends in public sector governance since the late 1990s notes that modes of governance have become more complex. In addition to the hierarchies and markets of the 1980s and 1990s, network arrangements were identified. Increased system complexity renders hierarchical modes inadequate over time [8,9]. There is a need to engage in network modes of governance with partners in a horizontal network to bring stakeholders together for a common end. Health governance internationally became more complex, with both hierarchical and network modes of governance explicitly represented within single public systems [8]. Barnett notes that, while there has been a decline in market arrangements, there is a persistence of hierarchical forms and greater presence of organisational (and community) networks. Tuohy [9] points out that, with increasing complexity of technology and relationships, traditional vertical directives may no longer be adequate and governments themselves may need a wider repertoire of skills, including negotiation and persuasion, to manage interdependent organisations and networks. In conceptualising the state- industry- sick fund network, I draw upon Rhodes [10,11] who argued for a definition which identifies governance as referring to ‘self-organising, inter-organisational networks’ and which contains four features:

■ interdependence between organisations;

■ continuing interactions between members of the network;

■ game-like interrelationships and interactivities, that are rooted in trust and regulated by rules of the game negotiated and agreed by network participants.

■ a large degree of autonomy from the state.

Governance and coordination are nouns, and an analysis that deploys these terms can sneak in an implicit assumption that some sort of governance and coordination has been achieved. However, the achievement of some requisite level of governance and/or coordination in different settings cannot be taken for granted. Rather than assuming the achievement of governance, and asking what type(s) are to be found, Tenbensel [12] suggested that analysis of multiple modes can gain more traction by focusing primarily on attempts to steer in the direction of any perceived ideal types of governance. In order to bring the political context into the foreground, it is useful to think of each of the modes of steering as contingent upon the mobilisation of particular types of power and knowledge resources. The various modes of governance and steering each require a different constellation of power and knowledge. Therefore, shifts of emphasis from one mode of steering to another require the gathering of different types of knowledge and the deployment of different power resources.

In this paper I try to capture and characterise the shifting modes of governance in the Israeli health and pharmaceutical sectors. I identify a shift from an unregulated market arrangement in the pre-NHI era that shifted to a mainly hierarchical mode of governance with the establishment of the NHI funding reform of 1995, and which continued through the early NHI "transition" years from 1995–1998. In the decade and a half since there has been a long-term shift from the mainly hierarchical mode to a mixture of hierarchical and horizontal modes of governance with the characteristic of a looser, more dispersed policy network.

To understand how and why a policy network operates, it is necessary to understand the unwritten constitution, which guides the behaviour of government, industry and sick funds towards each other and influences the strategic deployment of their resources. Some of the rules can be distinguished by what these actors say or write or believe the rules to be. Other rules can be inferred from their conduct to each other. Although there are apparently no regular formal consultative arrangements between the government and industry groups in Israel, there are many informal exchanges, some of which take place at the highest levels in the Ministry of Health [MOH] and other government ministries (Treasury, Industry, and Justice).

To understand better the shaping of the governance of the industry and the sick funds (the pharmaceutical "sector") over the last two decades I also draw on some of the theoretical concepts used by Abraham [1], in his investigation of the governance of the industry in the context of the UK National Health System (NHS) since the latter’s creation. In particular I draw upon the concept of countervailing powers [13,14] to corporate bias [15]. The theory of countervailing powers focuses on the role played by a range of actors and the pressure they exert, or fail to exert, to achieve or to block a dominant party’s position. It has recently been applied to understand the rapid growth of medicine use and the governance of the pharmaceutical industry [16,17].

## The Israel case

This section begins with a description of the Israeli political economy context focusing on public policy and the budgetary framework. This is followed by an analysis of governance in the Israeli pharmaceutical sector within the context of health insurance reform, referring to the shifting modes of governance and the multi-levelness of policy making and governance. The section then focuses on governance arrangements in the management of the NHI drug formulary (basket), in order to reflect on how well pharmaceutical governance has fared in the previous two decades and how the state has dealt with and resolved conflicting interests in the key areas of reimbursement and pricing. The section concludes with a comparison of the governance and institutional approaches taken by Israel and New Zealand, which has a central pharmaceutical management agency in a single payer NHS system.

### Political economy and public policy context

Prior to NHI, the health care system was plagued by a number of problems, the most relevant to this paper were financial deficits and lack of clarity regarding entitlements. The NHI Law was the main policy response to these problems [18]. Three related factors that are key to an understanding of the pharmaceutical sector in Israel and its development are the role of central government, the nature of the NHI reform and the reform’s impact on the pharmaceutical sector.

Central government dominates Israel’s political economy and the formation of public policy, with the Treasury being traditionally very influential in policy making. There has been a long-term central government focus on fiscal consolidation, containing public expenditures in all civilian areas including health, starting from around 1985 (Economic Recovery Programme). This was bolstered by the country’s financial and political leadership for most of the period since 1996. In the 1990s in particular the Treasury became the "policeman" of the public services, vetoing potentially costly reforms of state agencies and putting obstacles in the way of funding costly legislation. In Israel’s policy-making network, economic thinking has become preeminent in social welfare policy and ideology, particularly since the 1990s. In Israel, as in other countries, social policy took on a harder, neo-liberal edge to match the deregulation policies of the late 1980s. Often a gap arose between promises embedded in much of Israel’s welfare legislation and its actual implementation [19].

In 1995 the NHI Law went into effect, creating compulsory health insurance for all Israeli residents by means of one of 4 existing sick funds of their choice. The main goals of the law were to provide universal health coverage; spell out residents’ rights to a basic package of health services; promote increased equity; assure the solvency of the healthcare system; and give residents greater freedom of choice among sick funds. A key plank of an earlier reform proposal not incorporated into the NHI law, which remains on the agenda till this day, was to absolve the MoH of operational responsibility for the provision of health services, thereby allowing MoH to devote more effort to monitoring and regulating the system.

Although the 1995 NHI reform was a step forward in the social welfare sense, with Israel catching up with those of welfare state systems elsewhere, it was also a funding reform giving the government for the first time clear budgetary control of the health system. As implemented, a main thrust of the NHI reform was to enhance central government’s control over sick fund expenses in order to constrain public expenditures [20]. Some argue that government involvement in providing financial support to publicly-financed health services has continually been eroded during this period. As is the case with basic and comprehensive welfare legislation, the NHI Law intrinsically affords substantial leeway for bureaucratic or procedural judgement in its implementation by the MOH and the sick funds [19].

In general, Israeli public policy has been influenced by the paradigm of new public management (NPM). In the health sphere, since implementation of NHI in 1995, Israel has deployed a fairly comprehensive version of regulated competition for health care services, with open enrolment for citizens in four regulated, capitated sick funds. Perhaps the most challenging aspect of the regulated competition model, mandating and updating a standard basket of health services, has been carried out [21].

In the pharmaceutical policy domain, since the early 1990s and even before the introduction of NHI law, government in Israel had become increasingly active. Not only was it more active than in the past, but arguably for most of the period government has also been more active in the pharmaceutical domain than in most other areas of health policy. This has particularly been the case following the introduction of NHI in 1995, which cast the government, especially the MOH and the Treasury, in a much more central role as regulator in the pharmaceutical policy domain. In the early days, as now, a major policy concern has been the relentless growth in expenditure on prescribed medicines as a stream of new and costly therapies came onto the market. However, it was only with the establishment of NHI and the health services basket in the mid-1990s, that the cost of medicines and the issue of ensuring affordable access really became a focus of concerted public policy attention. With the government commitment under NHI to guarantee universal access to a defined basket of health services within a defined budget, prescription medicines were caught up in the emerging financial arrangements underpinning public health services in Israel. Public uproar over failure to update the basket and Treasury efforts to leave the determination of benefits to the sick funds, led in 1998 to a unique government involvement followed by a (near-total) commitment since then to provide an extra budget for annual update of the national basket of health services and the reimbursement of new drugs. At the same time the Treasury initiated legislation to formalize co-payments for services covered in the basket and this was approved by parliament.

### From market (pre-NHI) to hierarchical (since NHI) mode of governance

Before 1995, in the pre-NHI period, a market-based mode of governance was dominant, characterised by non-regulated competition between the sick funds. Traditionally in this period the MOH shared decision-making powers with the Clalit sick fund, mainly because of its dominance (then a 75-80% share of overall sick fund membership) and its political ties with the Histadrut (labour union) and Labour governments. The Treasury was also a powerful co-player but in this era the financial accountability mechanisms were loose.

As noted above, the increasing financial instability of the health system in the 1980s and early 1990s, particularly in the Clalit sick fund, highlighted the need for a more managed and regulated mode of governance. The NHI health reform of 1995 drew on the rhetoric of managed competition, but rather than fostering competition, the main thrust of the NHI reforms was to enhance central government control over sick fund expenses in order to constrain government expenditures [20]. The result was a dependence of sick funds on central government funding and a more transparent hierarchy of Treasury over MOH and of MOH over sick funds. In the pre-NHI era the boundary between policy and service provision was often blurred; in the NHI era this boundary became clearer, with government responsible for policy and the sick funds for service provision. The introduction of a clear budgetary framework for the NHI and the public health care system forced sick funds to adopt better management tools in order to run more efficiently (and possibly more effectively). The government increased its regulatory powers over the sick funds, particularly over Clalit. The voice of the sick funds became more expressed in operational management and implementation of policies decided at the State level. Clalit (and the other sick funds) remained a partner in policy negotiations, but Clalit no longer had veto power. Till this day the sick funds are not organised into a formal association which jointly and legally represents their interests and those of their members vis-a-vis government and regulators. This is opposed by both MOH and the Treasury - formally on antitrust considerations so as, it is claimed, to protect patient rights.

The introduction of NHI also promoted the government, that is, both MOH and the Treasury, to a much more central and active role as regulator in the drug policy domain. Previously, it was limited to medicines registration and price control. The state via the NHI had now become responsible also for the issue of universal access to a defined basket of health care services. In response to government concerns about equity and access there evolved greater MOH control of pharmaceutical services. Central policy and guidelines were introduced that were to underpin government desire for consistency of access and services, and these were maintained by mechanisms such as national policies mainly administered by the MoH’s Medical Administration and budgetary and financial accountability provisions carried out by the MoH’s Division of Supervision and Control of Sick funds.

### Mixed modes of governance (hierarchical and network) in the NHI era

In the NHI era Israel appears to have structured regulatory reform to protect valued institutional arrangements such as the sick funds whilst at the same time creating and then maintaining critical government capacities. Government has been concerned to preserve, not to destabilise or to significantly restrict, freedom of institutional capacities and competencies. The NHI funding reform was characterised by a determination and commitment to keep to the existing institutional configuration. Government has taken care that the reform has not affected the autonomy of the sick funds to keep and develop their own organisation models. The key characteristic of the loosely dispersed pharmaceutical policy network is that there is (clear) direct government control coupled with a decentralised policy implementation process, which can apply a commercial market management approach. Government leaves the sick funds substantial commercial freedom to negotiate with industry and pharmacies. Although within the framework of NHI there are no budgeting goals for pharmaceutical expenditures, this has allowed the government to succeed in fulfilling its core objective of containing pharmaceutical costs. Sick funds apply formulary management practices that include: specifying which of the available reimbursable products or brands may be prescribed; securing price discounts for drugs purchased; and where appropriate specifying conditions under which a physician may prescribe certain drugs for patients.

At the same time as protecting institutional arrangements, there has been also a rise in government technical competency which has brought reforms in key aspects of pharmaceutical policy, for example encouragement of the provision and use of generics to reduce costs and introduction of international (external) price referencing. The new national policy equilibrium required major change in the MOH. In addition to its traditional drug registration (market authorisation) and pricing control activities it has had to create new technical infrastructures (scientific, technical and economic), including health technology assessment (HTA) and the use of evidence-based medicine in reimbursement and associated national prescribing guidelines; supervision, mainly financial, of sick funds’ activities.

### NHI and the rationalization of pharmaceutical provision

Prior to the introduction of NHI in 1995, there was a lack of a clear national policy regarding reimbursement and priority setting for access to new drugs and technologies. Each sick fund had its own formulary. Decision making on what would be included in the formularies was mainly the outcome of negotiations between each of the sick funds and the commercial sponsor of each new drug. Sick fund formularies were not widely distributed and guidelines regarding their formation or update were not publicly available or discussed. In the pre-NHI era, priority setting by the private sick funds took place within a competitive insurer market that was not regulated or subject to government supervision. The drug formulary could be used by sick funds, if they wished, to select against high–risk patients. Industry’s efforts to attain formulary inclusion were mainly addressed at top sick fund management as well as at opinion and thought leaders in the medical profession working in sick funds and in hospitals. Connections between sponsors and members of the early policy elite (Clalit sick fund–Histadrut labour union-Labour Party) could be used to overcome problems or resistance. In the absence of strong budgetary control by the government over the sick funds at that time and in the context of a competitive sick fund market, sick funds had little incentive to exclude new drugs and technologies; critically this applied to the dominant sick fund Clalit which was losing market share. The "bill" in any case would be picked up by government which eventually had to bail out the Clalit on more than one occasion. In 1994 it was the Clalit’s dire financial situation that led to the establishment of the NHI.

The NHI basket of services specifies procedures and drugs and indications for use of these services. Although there is a common list of drugs to be covered under NHI, sick funds operate their own formularies. NHI gives parliament the right to remove items from the basket, and to add items on condition that budgets are made available by the government (implying agreement between the MOH and treasury) to cover the anticipated costs [21].

During the transition period of 1995–8 - whilst NHI was already in place but before the precedence of extra funding for new drugs and the establishment of national HTA, leading local companies succeeded in getting new expensive treatments for multiple sclerosis added to the basket. This was achieved in spite of an internal MOH economic assessment that spelled out the low cost-effectiveness of these treatments, which at that time were unproven in terms of efficacy, and in spite of the substantial budgetary implication of including them. In addition to the strenuous lobbying of these companies, pressure was also generated by claims in the courts mainly against the MOH and the sick funds for access to these treatments. The sick funds, which were formally absent from the closed debate within the MOH on this issue, were left to fund these treatments.

In 1998 a Public Committee was established for the first time in order to consider the addition of new services to the basket, with the Treasury appearing (with hindsight) to provide an annual increment of about 1% to the known cost of the basket for this purpose. The list of services, mainly pharmaceuticals, seeking inclusion in the basket far exceeds the available funding. The creation of this MOH-appointed Committee was designed, perhaps, to provide insulation for the MOH and government. The committee meets several times over a few months each year in order to discuss technologies which have been selected and ranked for prioritisation earlier in the year by internal non-transparent MoH procedures.

The update process in Israel is unique. In most of the Western world the inclusion of each specific technology is assessed sequentially, within the context of the annual health services budget. In contrast, in Israel, assessment of hundreds of technologies (ca 400 annually, a majority of which are resubmissions) competing with each other are carried out at the same time. The process of updating the basket essentially involves choosing between drugs that are not alternatives for treating the same clinical problem, but which are competing candidates for the extra monies determined and budgeted for this purpose. A major consideration in decision making is centered on the highly uncertain assessment of budget impact in the short to medium term. This approach tends to marginalise, at least in the public space, the key issue of what is the therapeutic advantage (marginal clinical effectiveness in relation to more established medications) of a candidate drug and of the quality of evidence to support it. The process begins with a ranking of potential new services, based on HTAs performed internally by the MOH and which is presented in summary form by it to the committee. The committee, which is made up of physicians, MOH representatives, a representative of each of the four sick funds, Treasury representative(s), and public representatives (2–3 with backgrounds in ethics, religion and law) then deliberates based on various ethical, economic and social criteria in order to arrive at a final ranking and a decision as to which services will be included within the available extra budget. Patient organisations and industry are not represented directly on the committee. There is lack of transparency and public access is limited to invited media. It is virtually impossible to follow the proceedings, particularly at the level of a specific technology. The media could be considered as a potential countervailing force in a debate regarding funding decision. However this potential is not realised; apart from the enormous complexity of following committee discussions, this potential is weakened due to the media’s underlying message that the NHI basket is basic and insufficient and that more funding is the solution.

In notable cases involving cancer drugs, such as Herceptin (trastuzumab) and Avastin (bevacizumab), pressure exerted by lobby groups and politicians overcame, apparently, the inclination of the committee based on HTA [21]. Moreover, the level of funding varies substantially from year to year, and the decisions of the committee are subject to Cabinet approval, such that the process [22], according to Chinitz [21], "remains a mix, sometimes unstable but still impressive, of science and politics [22-24]".

### Governance arrangements in reimbursement and pricing

In the NHI era, government has become a stronger part of an inter-organisational network of pharmaceutical governance consisting of key self-organised participants, industry and sick funds, groups that accept government authority within the network of governance. Survival of the network requires management of a range of difficult and politically volatile stakeholder relationships. Negotiation and accommodation, flexibility and trusting relationships are requisites of this network and help to ensure its survival. In the increasing complexity of reimbursement decision making since 1998, the role of these stakeholders has been strengthened. Sick funds have privileged access to and influence over the state in funding decisions. With access to HTA within the MOH and a representative of each sick fund on the committee (and also on its more exclusive key economic sub-committee) they are able to influence which drugs get added to the basket and under what conditions and restrictions.

The presence of sick funds helps to keep reimbursement decisions at a distance from the political arena. For instance, the government reimbursement process itself does not get involved in cases involving exceptional circumstances; such cases fall under the discretion of the sick funds which have had to set up their own "exception" committees [25].

As the MOH’s HTA and the committee cannot hold all the required competencies and information internally, they require the technical support, information and expertise provided by stakeholders; the involvement of industry (the main proposer and provider of HTAs) and sick funds helps to guarantee the legitimacy of the final assessment and helps to prevent conflict after the technologies have entered the market.

The drug industry has no formal access to the decision-making process in the committee and there is no appeal process. This lack of access is not a crucial matter for the local generic-based industry (see Appendix), as most submissions are made by multinationals and their local representatives. Nevertheless, the survival of the network also requires keeping on-side two influential stakeholders – the research-based multinational pharmaceutical industry and the medical profession. This has meant recognising innovation and managing the tension this creates vis-à-vis cost control objectives. Keeping both stakeholders on-side was facilitated by the extra budgets allocated for the annual updating of the drug basket. There are also several features of the process for updating the list which indicate that government wishes to avoid confrontation with "Big Pharma". These include: no prior price negotiations (thus implicitly delinking price premium with degree of innovation such as therapeutic advantage); not stating the grounds for non-inclusion; and automatic resubmission in the next cycle without having to address issues raised earlier (such as high prices and inadequate evidence base). The process was set up in the period when the government had to defend its policy regarding patent law amendments favouring the Israeli generic industry from strident criticism particularly in the USA capital, and the threat of sanctions from "Big Pharma".

In a comparative analysis of institutional design in several countries including Israel, Maor [26] classified national drug reimbursement agencies based on the level of independence of the reimbursement mechanism and the quality of drug evaluation. Dividing these mechanisms into four model types- adversarial, near-adversarial, trust and near- trust - Maor suggested that each model represents a distinct way in which governments can use institutional devices to manage the risks related to agency reputation. He concluded that Israel’s reimbursement agency is a classic example of a "trust" model which he characterised as being less independent from government ministries and as having less demanding standards of drug evaluation. In Israel the guidelines for clinical and cost- effectiveness evaluation do not represent a demand that companies demonstrate comparative therapeutic advantage. There is no indication of the preferred type of study, type of participant, type of interventions or type of outcome measures. The guidelines implicitly encourage the submission of the best available data or evidence without prescribing the type of evidence or data that is deemed acceptable [26].

Although the innovativeness of many new drugs is dubious, and many new drugs offer little health benefit (‘me-too’ drugs) beyond what is currently available, manufacturers will typically – and by business necessity must – claim innovation [27]. However, government involvement in the basket update process and in the prior and separate regulation of drug prices (by means of external reference pricing, ERP) does not constrain the prices of new products and thus it cannot be accused of not rewarding claims of innovation. New cancer drugs are often reimbursed earlier in Israel at prices similar or higher than in England and elsewhere [28]. Israel is prepared to recognise and pay the price of innovation, not least because of the political risk of not doing so in the case of a government-controlled non-independent reimbursement agency [26]. In the absence of price negotiation before a new drug is added to the basket the government is tacitly agreeing to high prices of new products in its funding decisions; such a policy can delay and sometimes prevent the inclusion of drugs. Although the market approval process is separate from the HTA and the reimbursement process, they are institutionally linked in as much as they are carried out by the same internal directorate in the MOH. In recent years there has been a pattern of the MOH issuing market approval in some cases only after the drug has become reimbursable.

With regard to pricing, a corporate bias has been dominant in the regulation of drug prices, permitting the industry to have privileged access and influence over government on this issue - access not afforded to other interest groups. ERP was introduced in 2001, linking drug prices to high-priced reference countries in Europe. At the same time ERP in Israel uniquely links the prices of generic drugs to original brand prices, and these listed prices- and not the discounted prices negotiated by the sick funds- form the basis on which co-payments levied by the sick funds, with MOH approval, are determined. Whereas, in the two decades before ERP was introduced, sick funds were a countervailing influence mobilised then to support government moves to price reform, they have since become aligned with the industry. Furthermore, in the competitive sick fund market there is little chance that sick funds will use their potential countervailing power against the reality of high prices being sought for moderate benefits by a drug’s commercial sponsor. In the absence of the MOH fulfilling its potential as a countervailing influence, and of any countervailing influence from the dominant sick funds in working against structural price distortions, the weakening of the regulation of price by corporate bias continues. The maintenance of the current price regulatory system since 2001 is testimony to the reluctance of the state to a price control system that better protects the interests of the NHI over and above the interests of the industry and the sick funds [29].

### Medical profession and disease-focused patient groups

Due to the non-transparency of the HTA process it is not clear to what extent submissions by sponsors are subject to independent review and critique as distinct from clarification from non-independent clinical specialists, sick funds and the sponsor. Independent scientific experts could act as a countervailing power to a sponsor that hopes to achieve a high ranking from the MOH’s HTA. On the other hand, in the absence of challenge by scientists and regulators to the power of manufacturers in funding decisions, this signifies some degree of corporate bias [30]. In the case of Herceptin lobbying and politicians overcame, apparently, the inclination of the professional committee based on HTA [21].

Enlarging the evidence-based medicine aspect, an integral part of HTA, does legitimise the cost-containing side of the reimbursement process as it is consistent with best practice and so keeps the medical community on-side. It also reflects an alignment of managerial and clinical rationalities, so that doctors are enrolled into a system of governance. In its management of the drug benefit scheme, the state is placed in opposition to the medical professions’ concerns about clinical autonomy. Those concerns encourage the industry to assimilate the medical profession as an ally in the industry’s quest to undermine those elements of the state (e.g., HTA) having countervailing effects. This, in addition to industry’s ability to mobilise alliances of organised groups of patients, to use courts, and to gain privileged access to government, helps to frustrate the state’s objectives, redirecting them more in line with industry interests.

Furthermore, industry’s creation of "assimilated allies" with an ideological assault on the consciousness of the medical profession - what doctors learn regarding diagnostic criteria, what they are told by opinion leaders in "continuing medical education", what they learn from biased medical journals and what they absorb from company representatives and advertisements - has promoted "pharmaceuticalisation" [1] inside and outside the NHI. (Outside the framework of the NHI basket these processes have thrived, aided by the widespread diffusion of more market-based supplementary insurance). Not all pharmaceuticalisation is contrary to the interests of health or the NHI, but some of it can be. The assault is so pervasive that it may be difficult for busy doctors to distinguish ideology about drugs from valid knowledge - all the more so if their patients are demanding particular drugs (recommended by industry-supported patient groups and the media). The growing "pharmaceuticalisation" and consumerism has expanded physicians’ prescribing horizon outside the NHI, making difficult the distinction between rational prescribing based on collective health priorities and promotional claims for the therapeutic value of new products. The twin processes of pharmaceuticalisation and consumerism have predominantly served to assimilate the potential countervailing powers of the medical profession and patients into allies of corporate bias in governance [1].

### Comparing governance and institutional approaches- Israel and New Zealand

This trend towards increasingly hierarchical modes of governance has also been the experience of New Zealand as well as a number of other small countries or jurisdictions, most notably those with a clear regional or local system of governance [8]. However unlike many Western developed countries, (including small-sized ones), central government in Israel has little local/regional control over most health services. In Israel the sick funds, rather than local or regional governments, manage the health services directly. One consequence of the lack of accountability to peripheral areas has been a growing level of health inequality in the NHI era.

Both Israel and New Zealand seem to have succeeded in cost-containment, but the two countries have taken different institutional approaches. In Israel there is a dispersed policy network in a multi - payer NHI system, whereas, in New Zealand, Pharmac was established as a central management agency in a single - payer NHS system. Pharmac’s key to success as a manager of a capped budget is considered to be due to the combination of governance arrangements independent of direct government control and a decision-making methodology that employs consistent and widely accepted assessment techniques, features that seem to be distinctive of comparable agencies elsewhere in the world [3]. In Israel, the government has left all matters related to actual drug pricing to the four sick funds. After a decision by the basket of services committee, sick funds are free to negotiate with the drug suppliers as well as to employ any supply-side strategies that they see fit. However aggressive sick fund supply-side strategies may be, the outcome, in terms of efficient government subsidies and hence low drug prices, according to Maor [26] does not come close to the situation whereby a national purchasing mechanism, such as Pharmac, employs similar strategies. Although a key measure undertaken in both countries is tendering for sole supply rights, examples of procedures undertaken by Pharmac but not by the Israeli reimbursement agency are:

■ Refusing to list a new drug for public financing unless it undercuts the price of the existing reference drugs.

■ Refusing to include a new drug if it is deemed that the market is sufficiently provided for.

On the demand side, Israel’s MOH, unlike Pharmac, has not needed to expand its role into demand management, leaving it to sick funds; prescribers’ education is mainly undertaken by the four sick funds that employ doctors.

## Discussion

This analysis suggests that a relatively complex mix of hierarchical and network modes of health governance has been successfully established over an extended period of time when flexibility is maintained through the implementation process. The system for defining and updating a standard basket of drugs has coped well with the challenge of managing a range of difficult and potentially volatile stakeholder relationships as well as distancing ministers from controversies of funding and listing decisions. Government has succeeded in containing drug costs whilst still maintaining a basket of reimbursable drugs that, in an international perspective, is technologically advanced.

On the other hand, it has coped less favourably in the area of financial access and equity to health care services for all citizens. Network arrangements in the Israel pharmaceutical sector also appear to have delayed the introduction of, or hindered, suitable accountability relationships, such as monitoring how sick funds have spent the extra funds allocated for basket update [31] and the extent of patients’ sharing of cost of drug treatments in the basket [32]. The lack of regional governance - there is no devolution of powers to regions in Israel’s health policy - means that there is no regional accountability on health care spending. The state has traditionally played, with little transparency and to the detriment of a more pluralistic access to decision making, an intermediary role between unavoidable corporate interests of industry and sick funds. Institutional and governance arrangements in Israel appear to limit the potential and/or incentive of the MOH and the sick funds to act as a countervailing power in subsidy and other decisions. In NHI, provision for real public accountability of pharmaceutical development and regulation remains bleak in a government-industry-sick fund complex that lacks transparency and a regulatory system whose public rights of access to information lag behind many other regimes in the West and other developed countries.

One of the effects of corporate bias has been a shift in policy away from regulation in the interests of patients and public health to prioritisation of the interests of the regulatees (industry, sick funds) instead e.g. prices, prescription co-payments as well as supplementary insurance outside the NHI. This focus on pharmaceuticals has diverted or delayed attention away from non-pharmaceutical needs e.g. dental health, long-term nursing care, mental health which were not included in the NHI basket of services. The "idea of innovation" has its success in that update of the basket was seen mainly technologically and pharmaceutically thus arguably delaying or preventing expansion of health services into these areas. These require establishing their own governance arrangements that do not have the unifying value of the idea of innovation that characterises governance of the pharmaceutical sector.

## Appendix: Pharmaceutical sector and industry context

Government has multifaceted and often conflicting roles in its relationship with the pharmaceutical domain: industry’s regulator (drug approvals and safety, prices); industry’s sponsor (promoting industrial competitiveness); and industry’s customer (government–owned hospitals). In addition, as the ultimate source of financing, it serves as a reimbursement agency (universal access and co-payments) and is deeply involved in supervision of the sick funds (budgets and efficiency). These multiple roles, combined with the complex set of interrelating regulations through which the regulatory process occurs, lead to a predisposition for negotiation in considering regulatory alternatives and trade-offs among policy areas. The common thread across the areas of product safety regulation, health policy and support of industry expansion is a trust-based exchange among a narrow range of stakeholders, with a blurring of public-private boundaries. As Lewis and Abraham have noted, in the more neo-liberal regulatory state that has developed, the State has become more responsive to and convergent with industrial interests; consequently, it has less to bargain with and about [33]. There has to be an informal atmosphere of trust between regulators and industry. With increasing government involvement and activism in health and drug policy in the NHI era, the extent of industry’s freedom from government action continues to be limited. On the other hand, industry, with a stake in all aspects of pharmaceutical policy and regulation, draws upon unique resources, such as expertise and lobbying capacity.

An examination of the very limited access to Israel’s process of pharmaceutical regulation (approvals and safety; prices) afforded to consumers, health professionals and other non-industry interests, reveals how limited pluralism is in the sector. Israel’s pharmaceutical regulation is much closer to corporatism, with all the trappings of social closure, than to pluralism. For example, in Israel expert advisory meetings on medicine regulatory matters are closed to outside scrutiny, in stark contrast to the FDA and closer to the EMEA model. By and large, the industry has been entrusted with regulatory compliance.

Pharmaceutical systems in different countries seem very different in terms of structure and organisation, and in most cases these differences stem from historical reasons where the weight of the local pharmaceutical industry plays a role. The Israeli pharmaceutical sector is characterised by the smallness of the domestic market and the presence of a large generic-based domestic industry that has become relatively influential in relation to the research-based multinational pharmaceutical industry. Subsidiaries of research-based companies were established relatively late in Israel, mainly during the decade starting from the mid-1990s. The influence of multinational pharmaceutical companies derives ultimately from their international mobility. In Israel their influence is in any case limited as they confine themselves mainly to marketing activities: they have no large-scale investments in the country, focusing mainly on small-scale clinical trials and the search for and acquisition of usually early-phase health care technologies.

Pharmaceutical industry activity in Israel, for much of its history, consisted of secondary formulation and packaging and importing activities, with limited capacity to influence policy and to enhance national policies aimed at supporting it. Since the 1990s the sectoriality of the drug industry operating in Israel became more clear–cut between local manufacturers, mainly of generics, and local subsidiaries of research-based multinational industry (involved in importing and marketing). This made it easier for regulators to identify and align with the core interests of the local generic-based pharmaceutical industry which has also become a positive contributor to the national balance of trade. Thus one would expect that some development of polices in the pharmaceutical sector would reflect a "tailoring" of policies suitable to this generic-based local industry.

As the political economic context changed, governance of the Israel pharmaceutical sector has evolved to a distinctive neo-corporate mode of negotiation and accommodation. Regulatory "hostility" seen in the past (such as the lag of several years in approval of a new drug [34]), has been replaced by a more sympathetic environment with pharmaceutical policy increasingly formulated within a ‘state-capital’ partnership. Industry has steadily gained greater access to the government agencies that shape pharmaceutical policy, increasing its opportunities to interact with decision makers and pressing forward its ideas and claims. The partnership approach has resulted in a number of significant government initiatives explicitly aimed at meeting the interests and concerns of industry. Most significant were major Patent Law reforms in 1995 and 2008, which were explicit government recognition of the need to provide a supportive environment for the generic industry as one of the larger industrial activities in Israel. Another has been the preferential tax arrangements given to national export champions such as that provided most generously to Israel’s leading pharmaceutical exporter.

The global pharmaceutical enterprise has flourished, and research-based manufacturers – acting both individually and through organised, transnational business networks – have gained considerable political influence within national and supra-national organisations [27]. If once neglected by Israeli regulators [34], the interests of the multinational globally integrated pharmaceutical industry have also become impossible to ignore. In most countries, strong government commitment to cost-containment has the potential to create a tense relationship with industry. In Israel, because of the generic-based manufacturing nature of the local industry there is arguably less tension than might otherwise be the case. The Israel government can get closer to the pharmaceutical industry and still retain its autonomy and authority, and allow sick fund monopsony power to constrain drug expenditure. In general, the pharmaceutical industry uses the argument that cost containment is ‘toxic’ to the idea of innovation and thus to a country’s prospect of economic growth. But in Israel, the country’s economic growth is perceived to be also tied to the wellbeing of a strong generic (export-based) industry.

## Competing interests

The author declares there are no competing interests.

## Authors’ information

Philip Sax is the publishing editor of PHARMA Israel Drug Bulletin, an independent source of drug information and analysis in the area of pharmaceutical policy and economics.
